# 

**Published:** 1962-04

**Authors:** 


					t- O b M
4
,/f
THE
MEDICAL JOURNAL
OF THE
SOUTH-WEST
Incorporating
The Bristol Medico-Chirurgica1 Journal
Ad^4-I?W62
VOL. 77 (iij No. 284 Price 4J-

				

